# Lumbosacral spondylodiscitis following superior hypogastric nerve block performed during uterine artery embolization: a case report

**DOI:** 10.1186/s42155-026-00719-2

**Published:** 2026-06-12

**Authors:** Hamidreza Rouientan, Dina Seyedi, Shahram Akhlaghpoor

**Affiliations:** Department of Interventional Radiology, Pardis Noor Medical Imaging and Cancer Center, Pardisnoor Niloo, No. 30, Farokhi Yazdi Ave, Pasdaran, Tehran, Iran

**Keywords:** Uterine artery embolization, Superior hypogastric nerve block, Spondylodiscitis, Embolization

## Abstract

**Background:**

Uterine artery embolization (UAE) is a minimally invasive, uterus-preserving treatment for symptomatic uterine fibroids with a low infection rate, and post-procedural infections are typically confined to the pelvis. Extra-pelvic deep infections are exceptionally rare. We describe an unusual delayed presentation of lumbosacral spondylodiscitis following an otherwise uncomplicated UAE.

**Case presentation:**

A 30-year-old woman with symptomatic uterine fibroids underwent bilateral UAE. Embolization was performed with 700–900 μm microspheres to angiographic stasis, with adjunct superior hypogastric nerve block (SHNB) for analgesia via an anterior suprapubic approach. Recovery was initially uneventful; however, new low back pain developed approximately 1 week post-procedure. At day 20, inflammatory markers were mildly elevated with a normal white blood cell count, and lumbar magnetic resonance imaging (MRI) demonstrated no abnormality. Despite conservative management, pain progressively worsened with functional limitation. Repeat MRI at 7 weeks revealed L5–S1 spondylodiscitis. After antibiotic therapy, follow-up MRI at 11 weeks demonstrated progression with abscess formation, prompting referral to neurosurgery for further management.

**Conclusion:**

Persistent or progressive back pain after UAE, particularly when inflammatory markers rise, warrants reassessment and consideration of spondylodiscitis. Early MRI may be normal; repeat MRI is appropriate when symptoms persist or worsen. This case highlights spondylodiscitis as a rare but serious complication in the procedural setting, with SHNB representing a possible contributory mechanism.

## Introduction

Uterine artery embolization (UAE) is a minimally invasive procedure used to treat symptomatic uterine fibroids, offering a uterus-preserving alternative when medical management fails. UAE is generally considered safe, with studies showing shorter hospital stays, quicker recovery, and fewer major complications compared to surgical options [1–3]. The post-procedural course typically involves transient, self-limiting events, including vaginal discharge and symptoms associated with the post-embolization syndrome [4].

While post-procedural infection is a recognized complication, with a reported incidence of 2–3%, most infections remain localized and respond to antimicrobial therapy [5]. It is crucial to distinguish common localized pelvic infections from the rare event of deep-seated infections affecting extra-pelvic structures.

We report an unprecedented case of spondylodiscitis, diagnosed several weeks following an otherwise technically successful UAE.

## Case presentation

A 30-year-old woman presented with symptomatic uterine fibroids manifesting as heavy menstrual bleeding, pelvic pressure, dull pelvic pain, and frequent constipation. After comprehensive evaluation and counselling regarding treatment options, the patient elected to undergo UAE.

The UAE procedure was performed via the right common femoral artery under conscious sedation. After placement of a 6-Fr vascular sheath, pelvic angiography was performed using a 5-Fr catheter positioned in the internal iliac artery (Impress, Merit Medical Systems, UT, USA), followed by selective catheterization of each uterine artery with a 2.8-Fr microcatheter (Merit Maestro, Merit Medical Systems, UT, USA); the catheter tip was advanced into the horizontal segment of the uterine artery, distal to relevant non-target branches, to optimize the safety and effectiveness of embolization.

**Fig. 1:**
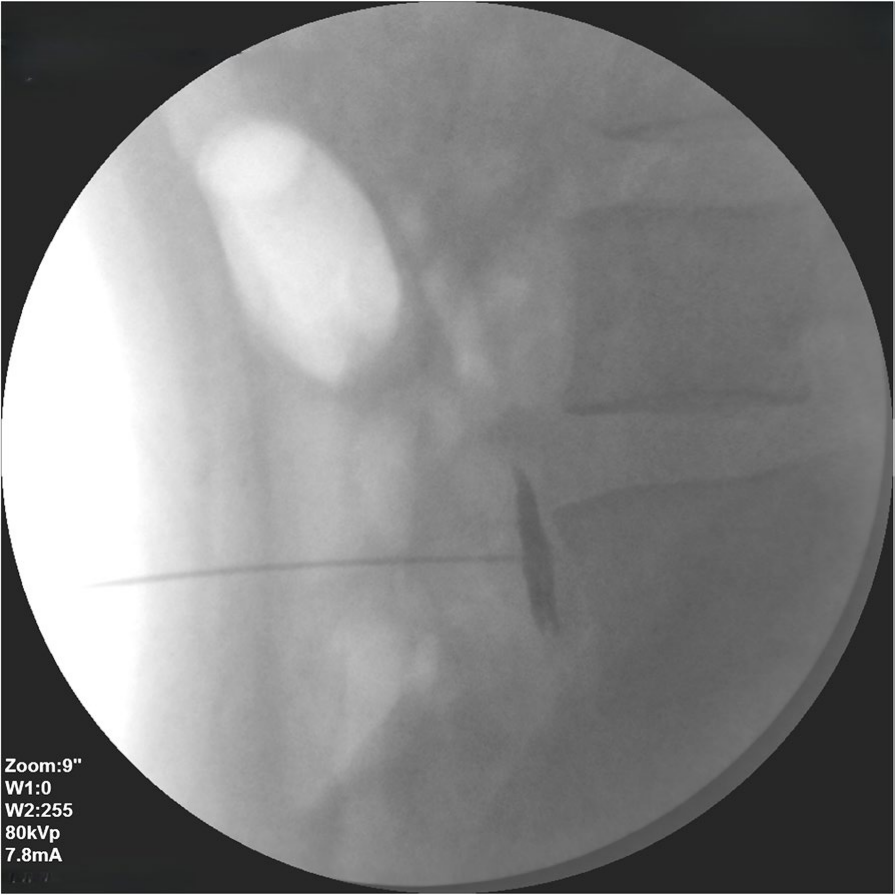
Fluoroscopic confirmation of needle position during superior hypogastric nerve block

Embolization was achieved using 700–900-μm Embosphere Microspheres. The embolization endpoint was stasis of contrast within the main uterine artery. A superior hypogastric nerve block (SHNB) was performed under fluoroscopic guidance via an anterior suprapubic approach. A 22-G Chiba needle (Cook Medical, Bloomington, IN, USA) was advanced percutaneously toward the prevertebral space. Needle advancement was performed initially under a true anteroposterior view, followed by lateral confirmation. The target was the anterior aspect of the vertebral body below the aortic bifurcation, while avoiding the intervertebral disc. Correct needle position was confirmed using a small test injection (approximately 2–3 mL) of diluted iodinated contrast (Fig. 1). On the lateral view, contrast was expected to remain confined to the prevertebral retroperitoneal space. On the anteroposterior view, symmetric spread across the midline was confirmed. The block was then performed by injecting 4 mL of 0.5% bupivacaine and 2 mL of triamcinolone acetonide, with fluoroscopic confirmation of symmetric midline spread, consistent with successful block. The immediate post-procedural period was uneventful, with appropriate pain control and no evidence of adverse events. Prophylactic antibiotics included ceftriaxone 1 g administered prior to the procedure, and doxycycline 100 mg orally twice daily was prescribed for 5 days post-procedure. The patient was discharged on the same day with standard post-embolization care instructions.

**Fig. 2:**
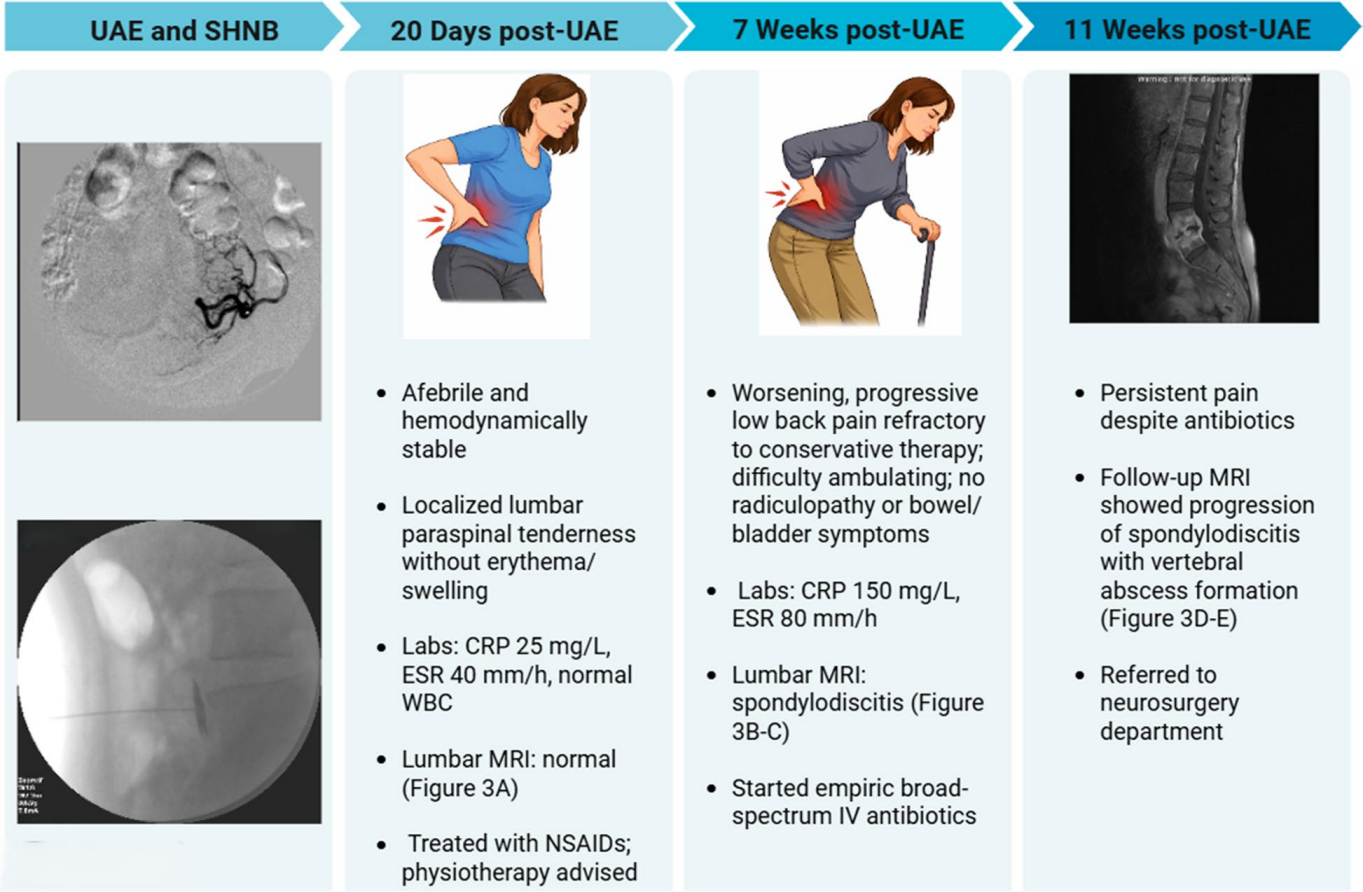
Timeline summarizing the patient’s clinical course following uterine artery embolization

**Fig. 3:**
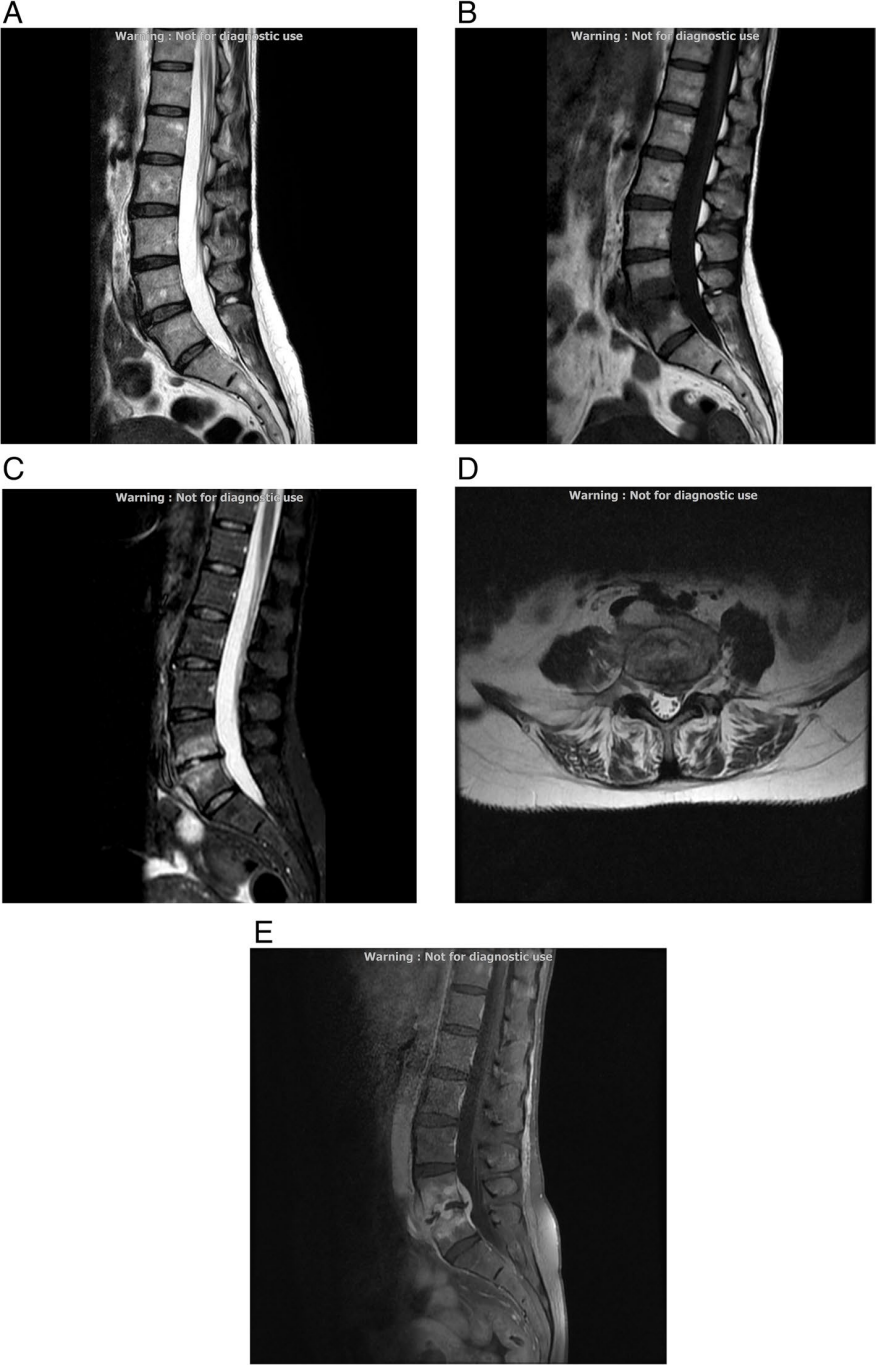
**A** Sagittal T2-weighted MRI of the lumbar spine demonstrates preserved vertebral body heights and intervertebral disc spaces, without endplate marrow edema or paravertebral/epidural fluid collection. **B**, **C** Sagittal lumbar spine MRI demonstrating interval signal abnormality at L5–S1. **B** Sagittal T1-weighted image shows low T1 marrow signal involving the adjacent vertebral endplates, with mild disc space narrowing/irregular endplate margins. **C** Sagittal T2-weighted fat-suppressed image demonstrates hyperintense marrow edema and increased disc signal, without significant epidural fluid collection consistent with spondylodiscitis. **D**, **E** Follow-up lumbar spine MRI (11 weeks post-procedure) demonstrating progression of L5–S1 spondylodiscitis with extradural collection. **D** Axial T2-weighted image shows abnormal high signal involving the anterior paravertebral soft tissues, without marked central canal compromise. **E** Sagittal T1-weighted image demonstrates destructive change centered at L5–S1 with a focal low-signal cavity within the L5 vertebral body and surrounding abnormal marrow/adjacent soft-tissue inflammatory change, compatible with abscess formation

Twenty days post-procedure, the patient presented with mild lower back pain that had commenced approximately 1 week following the UAE. Laboratory tests showed mildly elevated inflammatory markers, though the white blood cell count was normal. As shown in Fig. 2, the timeline summarizes the temporal progression of clinical symptoms, inflammatory markers, and imaging findings following UAE. A lumbar magnetic resonance imaging (MRI) was performed, demonstrating no abnormalities (Fig. 3A).

Seven weeks post-procedure, the patient returned with worsening low back pain that had progressively intensified and was refractory to conservative management. She reported increasing difficulty with ambulation but no radiculopathy or bowel/bladder dysfunction. Repeat lumbar spine MRI revealed findings consistent with spondylodiscitis (Fig. 3B, C).

Despite antibiotic treatment (vancomycin 1 g every 12 h and ceftriaxone 2 g once daily for 2 weeks, followed by oral levofloxacin 750 mg daily for 3 weeks), the patient continued to experience persistent low back pain. At 11 weeks post-procedure, follow-up MRI demonstrated progression of the spondylodiscitis with development of abscess (Fig. 3D, E). Due to the complexity of the infection and presence of abscess formation, the patient was referred to the neurosurgery department for consideration of surgical intervention.

## Discussion

While UAE is a safe, minimally invasive procedure with a well-characterized complication profile [3, 4], the development of a deep-seated, extra-pelvic infection affecting the intervertebral disc several weeks post-procedure represents a highly unusual and clinically significant complication. Spondylodiscitis most often results from hematogenous seeding of the disc and adjacent vertebral endplates. Although uncommon, spondylodiscitis has been described following urologic interventions. The best documented bladder-associated entity is vertebral osteomyelitis/spondylodiscitis due to Mycobacterium bovis as a delayed complication of intravesical Bacillus Calmette–Guérin therapy for bladder cancer, with presentations reported months to years after treatment and, in some cases, associated paravertebral or epidural collections [6].

In the context of UAE, two mechanistic pathways are plausible. The first mechanism is transient bacteremia from uterine infection or necrotic fibroid tissue, with hematogenous spinal seeding [5]. The necrotic tissue within the fibroids acts as a septic focus, and the subsequent bacteremia likely involves the Batson’s vertebral venous plexus [7]. This plexus connects the deep pelvic veins directly to the vertebral column circulation, providing an easy, direct route to the intervertebral disc space. This valveless system allows retrograde flow of the bacteria from the pelvis to the lumbar spine, bypassing the systemic circulation and directly introducing the infection to the disc space. Prevention should target uterine and access-site bacteremia. A structured pre-procedure assessment is recommended, and UAE should be postponed if there are clinical features of active pelvic infection. Per SIR/CIRSE guidance, prophylactic antibiotics for UAE are reasonable, with cefazolin 1–2 g IV commonly suggested within 1 h prior to the procedure [8]. However, this recommendation is Class IIa/Level C-EO, indicating limited evidence and expert-opinion support rather than comparative trial data. Published UAE guidance has also noted that, given the limited data, antibiotic selection may be guided by local hospital policy [5]. In our institution, ceftriaxone was used according to local policy to provide coverage for pelvic and genital tract organisms potentially implicated in post-UAE infection. Further comparative studies are needed to determine the optimal prophylactic regimen for UAE. Routine post-procedure antibiotics are generally unnecessary when no additional risk factors exist, and available evidence has not shown significant reduction in infection rates with a routine post-UAE oral antibiotic course when all patients received pre-procedure cefazolin. In selected higher-risk patients, targeted post-procedure antibiotics can be used; specifically, the SIR/CIRSE recommends doxycycline 100 mg orally twice daily for 7 days in women with hydrosalpinx [5].

A second possible mechanism relates to the SHNB performed for analgesia in this case. In the UAE setting, SHNB has been reported as an effective adjunct and has not been associated with major block-related complications in randomized data [9]. In addition, some studies have explored the addition of corticosteroids to the SHNB injectate to prolong analgesia [10]. However, corticosteroids may exert local immunosuppressive effects, which could theoretically increase susceptibility to infection in tissues. This technique requires deep needle advancement toward the prevertebral space and the literature acknowledges a theoretical risk of visceral injury or contamination with enteric flora [11]. Although no immediate adverse event was documented in this case, an unrecognized bowel micro-injury during needle passage and seeding of micro-organisms via contamination of the needle tip could theoretically introduce enteric organisms. In this patient, the temporal association raises the possibility of procedure-related inoculation, but causality cannot be proven. For the SHNB, bowel and bladder preparation, Trendelenburg positioning, and use of a smaller-gauge Chiba needle are recommended to reduce the risk of visceral injury by allowing the viscera to fall away from the needle path.

This report has several limitations. First, microbiological confirmation was not obtained, as blood cultures and biopsy were not performed; therefore, the causative organism could not be identified. Second, long-term follow-up after referral to neurosurgery was not available, as our institution functions as an outpatient interventional radiology center.

## Conclusion

This case reinforces that new back pain after UAE should not be attributed to musculoskeletal strain or post-embolization syndrome without reassessment. Post-embolization syndrome usually occurs early after UAE. In contrast, persistent or progressive back pain, especially with fever or rising inflammatory markers, warrants evaluation for infection, including spondylodiscitis. Repeat MRI is appropriate when symptoms evolve or suspicion persists. Targeted antimicrobial therapy and multidisciplinary management are important to reduce the risk of neurologic compromise and the need for complex surgical intervention.

## Data Availability

All data generated or analyzed during this study are included in this published article.

## Abbreviations

UAE: Uterine artery embolization
SHNB: Superior hypogastric nerve block
MRI: Magnetic resonance imaging
